# Conservative Kidney Management in the Middle East and North Africa: A Comparative Study of Conflict and Nonconflict Settings

**DOI:** 10.2215/CJN.0000000900

**Published:** 2025-12-05

**Authors:** Akram Al-Makki, David Gunderman, Valerie Luyckx, Taha Hatab, Imed Helal, Mayssaa Hoteit, Rana Yamout, Ala Ali, Abdulhafid Shebani, Najib AbuOsba, Mohamed H. Sayegh, Krishna Allam, Sahar H. Koubar

**Affiliations:** 1 Indiana University Health Arnett, Lafayette, Indiana; 2 Indiana University School of Medicine-WL, West Lafayette, Indiana; 3 University Children's Hospital, University of Zurich, Zurich, Switzerland; 4 Department of Public and Global Health, Epidemiology, Biostatistics and Prevention Institute, University of Zurich, Zurich, Switzerland; 5 Renal Division, Brigham and Women's Hospital, Harvard Medical School, Boston, Massachusetts; 6 Department of Pediatrics and Child Health, University of Cape Town, Cape Town, South Africa; 7 Houston Methodist DeBakey Heart and Vascular Center, Houston, Texas; 8 Department of Nephrology and Internal Medicine, Charles Nicolle Hospital, Tunis, Tunisia; 9 Department of Internal Medicine, Bridgeport Hospital/Yale New Haven Health, Bridgeport, Connecticut; 10 Division of Palliative care, Medicine Department, American University of Beirut, Beirut, Lebanon; 11 Medical Department, Nephrology and Renal Transplantation Center, Baghdad, Iraq; 12 Nephrology Department, Tripoli University Hospital, Tripoli, Libya; 13 Division of Nephrology, University of Yemen, Sanaa, Yemen; 14 Faculty of Medicine, American University of Beirut, Beirut, Lebanon; 15 School of Medicine, University of Minnesota, Minneapolis, Minnesota; 16 Division of Nephrology and Hypertension, University of Minnesota, Minneapolis, Minnesota

**Keywords:** CKD, ESKD, ethics, CKD nondialysis, conservative management, palliative care, supportive care

## Abstract

Conflicts severely disrupt care for patients with kidney failure, often limiting access to dialysis. In these challenging settings, conservative kidney management (CKM) emerges as a compassionate alternative focused on quality of life and symptom relief. This study explores how conflict conditions shape nephrologists' perceptions and implementation of CKM across the Middle East and North Africa (MENA) region. A web-based survey was distributed across 17 Arabic-speaking MENA countries over 3 months in 2022. Responses were divided into two groups according to the country's conflict status. Of the 334 nephrologist respondents, 66 were practicing in conflict zones and 268 in nonconflict zones. Conflict zone respondents were more likely to work part-time and less likely to be female. Dialysis in conflict zones was more likely funded by charitable sources (odds ratio [OR], 4.22; 95% confidence interval [CI], 1.36 to 13.07). Compared with nonconflict zones, nephrologists in conflict areas reported significantly less access to palliative care (OR, 3.36; 95% CI, 1.3 to 8.68) and cited lack of CKM training and limited access to CKM programs as barriers to its implementation (OR, 2.59; 95% CI, 1.24 to 5.38 and OR, 3.42; 95% CI, 1.59 to 7.35; respectively). While overall awareness of CKM was similar, those in conflict zones expressed greater moral and religious discomfort with withholding dialysis. These findings underscore important ethical, operational, and access-related disparities in CKM delivery in conflict zones in the MENA region. Addressing these gaps through supportive ethical policies focusing on procedural and distributive justice, increased nephrologists' awareness and training, and enhanced efforts to strengthen palliative care services are essential tools for establishing CKM as a viable and ethical approach for patients in conflict-affected regions.

## Introduction

Armed conflicts can severely disrupt health care services in numerous ways, including severe shortages of medicines and essential supplies, the loss or destruction of health care facilities, and the exodus of medical personnel, resulting in critical shortage of health care resources for primary and secondary health care.^1^ Unlike natural disasters, these conflicts are typically prolonged, leading to a shift in priorities toward acute care and an erosion of quality of care. As a result, individuals with chronic diseases often face inadequate treatment, lack of follow-up, and poorer health outcomes.^2^

Patients with kidney failure (KF) who are undergoing dialysis represent a highly vulnerable population during conflicts. Their care can be disrupted and altered, resulting in a significant rise in morbidity and mortality.^3^ In war-torn areas, the limited availability of resources such as medical personnel, equipment, and KRT options (such as dialysis or transplant) forces health care providers to prioritize alternative, more achievable forms of care albeit with different therapeutic goals. In this regard, conservative kidney management (CKM) becomes a pragmatic and compassionate approach for managing KF when KRT is unavailable. This ensures that patients receive quality supportive care even in difficult circumstances, with the goal of preserving dignity, alleviating symptoms and enhancing quality of life when life-saving KRT is not possible. During conflicts, every effort should be exercised to ensure noninterruption of KRT services; however, when KRT is limited, the prudent use of scarce resources and the principle of nonabandonment of the patients needing KRT may require prioritization of symptom management and patient comfort over ongoing attempts to deliver highly suboptimal care or no care.^3^

CKM is defined as “Care for people with KF that focuses predominantly on providing Kidney Supportive Care to promote quality of life but does not include KRT.”^4^ CKM can be chosen through shared decision-making in its traditional form or emerge as a necessity driven by resource limitations—a situation referred to as “choice-restricted” CKM. In either case, CKM should not be mistaken for “no care” or “rationing of care.” Instead, it is an active patient-centered approach that involves holistic support across medical, psychologic, social, spiritual, dietary, and cultural domains and extends beyond individual patients to alleviate the burden on their families as well.^4–6^ In low-income settings, where KRT is limited, choice restricted CKM is reported in 58% of countries, highlighting that it is often the only viable option for many individuals with KF.^7^

Currently, nothing is known about the extent of CKM utilization in patients with advanced kidney disease in areas of conflict. This work builds on our previous study on CKM in the Middle East and North Africa (MENA) region, which described general barriers and perspectives across the region.^8^ In the current analysis, we aimed to explore how conflict status influences nephrologists' awareness, acceptance, and implementation of CKM programs in the MENA region, an area that has witnessed some of deadliest armed conflicts in the 21st century. We specifically focused on identifying ethical, operational, and access-related disparities between conflict and nonconflict settings and aimed to propose potential solutions and policy recommendations.

## Methods

### Study Population

This study represents a focused analysis derived from a broader project aimed at exploring nephrologists' perspectives and practices related to CKM in the MENA region. The overall survey findings have been published in a separate paper as well as a detailed methodology.^8^ A summarized methodology is presented here. Over the period of April–August 2022, we conducted a web-based survey using the Lime Survey platform.^9^ The survey was designed in accordance with the checklist for reporting results of internet e-surveys criteria for online surveys to ensure robust data quality and representativeness.^10^ This survey specifically targeted nephrologists practicing in the Arab speaking countries of the MENA region. The Arabic speaking countries of the MENA region compromise Algeria, Bahrain, Egypt, Palestine (Gaza and the West Bank), Iraq, Jordan, Kuwait, Lebanon, Libya, Morocco, Oman, Qatar, Saudi Arabia, Sudan, Syria, the United Arab Emirates, Tunisia, and Yemen. The identification and contact of individual nephrologists were facilitated through their respective local nephrology societies. This study was approved by the American University of Beirut Institutional Review Board. Confidentiality and privacy of the participants were assured. The electronic questionnaire was submitted anonymously.

### Survey Description

Each registered nephrologist received an initial email to complete the survey, followed by another reminder email in 1- and 2-month period. The questionnaire consisted of 40 questions. It comprised sections addressing participant demographics, characteristics of their dialysis facilities, patient profiles, knowledge, practices, attitudes toward CKM, and a case study. The survey was available in three languages: Arabic, English, and French (Supplemental Table 1). Each survey started with the definition of CKM, followed by signing an informed consent to participate.

### Statistical Methods

Responses were divided into two groups according to the country's conflict status. The areas of conflict included Iraq, Libya, Palestine (Gaza and West Bank), Syria, and Yemen.^11^ Categorical variables were reported as percentages. Continuous variables were reported as mean and SD if normally distributed and median and interquartile range if data were skewed. *P* values were considered significant if <0.05. Data were analyzed using SPSS (IBM Corp. [2017]. IBM SPSS Statistics for Windows. Armonk, NY: IBM Corp.) and R version 4.2.3. Univariate analysis comparing conflict versus nonconflict groups was conducted using *t* tests for numerical variables and chi-square tests for factor variables. To determine the independent effect of conflict status on nephrologist knowledge, attitudes, and practice, as well as answers to the case study questions, multivariable analyses were conducted with covariates selected *a priori*: sex, age, employment status, years in nephrology, facility location (urban/suburban/rural/other), facility setting (academic/private), funding entity for dialysis, and access to palliative care. These covariates were chosen for their clinical plausibility and prior evidence demonstrating their influence on physician knowledge and practice. Employment status, years in practice, and practice setting are known to shape individual knowledge and practice behaviors, and thus, adjusting for them helps reduce bias and improve the validity of our findings. For binary outcomes, logistic regression was used to determine significance and estimate odds ratios (ORs) with 95% confidence intervals (CIs). For multinomial outcomes, multinomial logistic regression was used, with the significance of predictors evaluated using likelihood ratio tests. Analyses were conducted using complete-case data, excluding responses with missing values in outcome, predictor, or covariate variables. To ensure sufficient cell sizes for robust model estimation, response levels with fewer than three observations for univariable models and fewer than four for multivariable models were excluded from analyses, and these levels were notated “Insuff data” in results tables. To assess multicollinearity among covariates, we confirmed that values of the degree-of-freedom-adjusted generalized variance inflation factor were <2.

## Results

**Figure 1:**
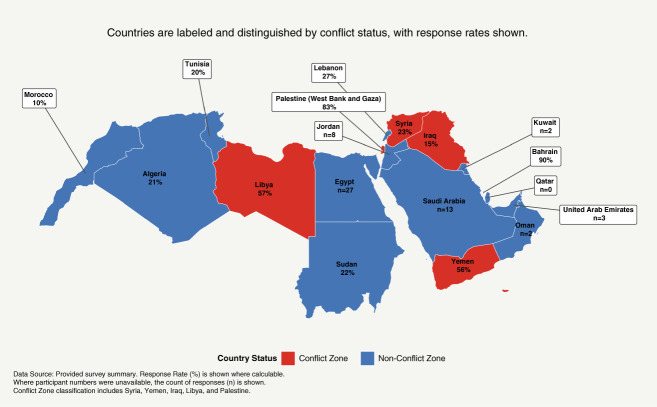
**Geographic distribution of survey respondents in the MENA region.** MENA, Middle East and North Africa.

We received responses from nephrologists practicing in 17 of the 18 Arabic-speaking MENA countries. We had no participants from Qatar. A total of 1240 potential participants received the survey. Three hundred and thirty-four responses were analyzed for this study: 66 responses from conflict areas and 268 from nonconflict areas. Two of the nephrology societies did not provide the total number of their members. Excluding these two societies, the survey participation rate was 40% in conflict zones and 24% in nonconflict zones (Figure 1). Table 1 presents the characteristics of the participating nephrologists as well as their facilities in conflict and nonconflict areas. A smaller proportion of female nephrologists practice in conflict zones compared with nonconflict zones (13% versus 87%; OR, 0.43; 95% CI, 0.24 to 0.76; *P* < 0.01). Part-time nephrologists are more prevalent in conflict zones as compared with those in nonconflict areas (OR, 3.18; 95% CI, 1.61 to 6.28; *P* < 0.001). More nephrologists in conflict zones tend to work in governmental settings (OR, 2.73; 95% CI, 1.15 to 6.49; *P* < 0.05). Funding for dialysis and palliative care in conflict areas is more likely to be from charity as compared with nonconflict zones (OR, 4.22; 95% CI, 1.36 to 13.07; *P* < 0.05 and 4.19; 95% CI, 1.21 to 14.55; *P* < 0.05, respectively). Fewer dialysis units in conflict areas had a social worker when compared with nonconflict areas (OR, 0.35; 95% CI, 0.19 to 0.66; *P* < 0.01).

**Table 1:** Baseline characteristics of the survey participants and their facilities in conflict and nonconflict areas^a^

Demographic Characteristics	Total, *N*	Nonconflict (*n*=268)	Conflict (*n*=66)
Age (yr), (*N*=334) mean±SD	43 [9.8]	44 (10.2)	42 (8.3)
		—	−1.71 (−4.37 to 0.94)
Sex (*N*=334, female)	166 (50)	144 (54)	22 (33)
		—	0.43 (0.24 to 0.76)^b^
**Current employment situation (*N*=334)**			
Have a full-time employment	276 (83)	233 (87)	43 (65)
		—	—
Have a part-time employment	46 (14)	29 (11)	17 (26)
		—	3.18 (1.61 to 6.28)^c^
Retired	5 (2)	5 (22)	0 (0.0)
		—	Insuff data
I prefer not to answer	7 (2)	1 (0.4)	6 (9)
		Insuff data	32.52 (3.82 to 276.99)^b^
**No. of years in nephrology practice after graduation (*N*=334)**			
<5	77 (23)	59 (22)	18 (27)
		—	—
5–10	102 (31)	78 (29)	24 (36)
		—	1.01 (0.5 to 2.03)
10–15	60 (18)	46 (17)	14 (21)
		—	1 (0.45 to 2.22)
15–20	34 (10)	31 (12)	3 (5)
		—	0.32 (0.09 to 1.16)
>20	61 (18)	54 (20)	7 (11)
		—	0.42 (0.16 to 1.10)
**Facility location (*N*=334)**			
Urban (city)	305 (91)	241 (90)	64 (97)
		—	—
Suburban	14 (4)	13 (5)	1 (2)
		—	Insuff data
Rural/village	15 (5)	14 (5)	1 (2)
		—	Insuff data
**Facility setting (*N*=332)**			
Academic	60 (18)	53 (20)	7 (11)
		—	—
Private	97 (29)	85 (32)	12 (19)
		—	1.07 (0.40 to 2.89)
Governmental	151 (46)	111 (41)	40 (63)
		—	2.73 (1.15 to 6.49)^d^
Military	6 (2)	6 (2)	0 (0.0)
		—	Insuff data
Nonprofit	8 (2)	6 (2)	2 (3)
		—	Insuff data
Other	10 (3)	7 (3)	3 (5)
		—	3.24 (0.68 to 15.52)
**Funding entity for dialysis (*N*=334)**			
Governmental	254 (76)	199 (74)	55 (83)
		—	—
Insurance	43 (13)	42 (16)	1 (2)
		—	Insuff data
Private (self-pay)	15 (5)	13 (5)	2 (3)
		—	Insuff data
Charity	13 (4)	6 (2)	7 (11)
		—	4.22 (1.36 to 13.07)^d^
Other	9 (3)	8 (3)	1 (2)
		—	Insuff data
**Funding entity for palliative cared (*N*=332)**			
Governmental	199 (60)	166 (62)	33 (50)
		—	—
Insurance	31 (9)	30 (11)	1 (2)
		—	Insuff data
Private (self-pay)	74 (22)	54 (20)	20 (30)
		—	1.86 (0.99 to 3.51)
Charity	11 (3)	6 (2)	5 (8)
		—	4.19 (1.21 to 14.55)^d^
Other	17 (5)	10 (4)	7 (11)
		—	3.52 (1.25 to 9.92)^d^
**Patients responsible for paying for their dialysis sessions (*N*=332)**			
Not at all	284 (85)	233 (88)	51 (77)
		—	—
Partially	43 (13)	30 (11.3)	13 (20)
		—	1.98 (0.97 to 4.06)
Fully	5 (2)	3 (1)	2 (3)
		—	Insuff data
Do you have a nephrology social worker in your dialysis unit? (*N*=331, yes)	129 (39)	115 (43)	14 (21)
		—	0.35 (0.19 to 0.66)^b^

a Each row presents the responses in conflict and nonconflict zones. Within each cell, the first line is count (%) or mean (SD) and the second line is univariable odds ratio (95% confidence interval) or difference of means (95% confidence interval). Odds ratios are in comparison with a baseline of nonconflict regions. For gender, “male” serves as the baseline, and only an odds ratio for “female” is listed. For multiple-choice questions, the first option is the baseline, and odds ratios are not provided for this choice. Insuff data mean that the count for that answer choice was too low to calculate an odds ratio.

b Significance levels=0.01.

c Significance levels=0.001.

d Significance levels=0.05.

**Figure 2:**
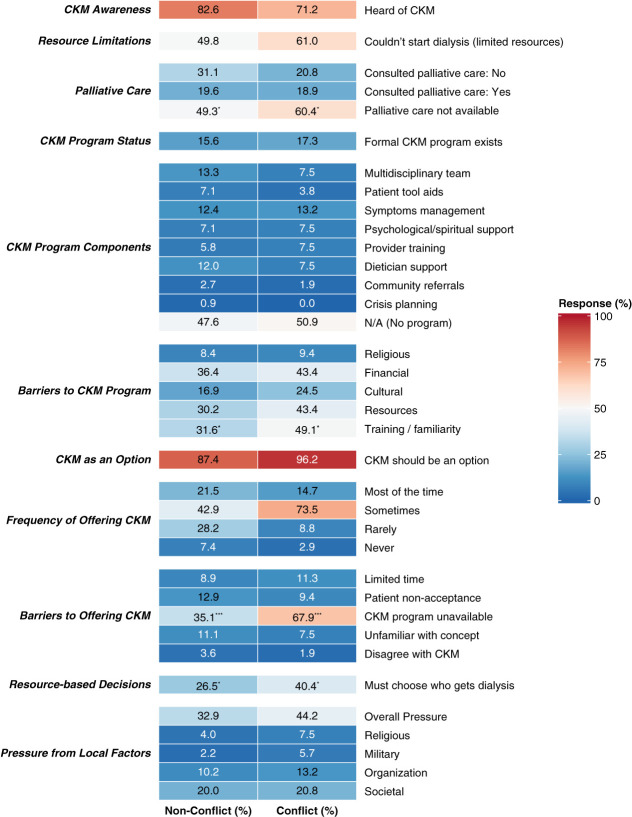
**Heat map of CKM perceptions among nephrologists in conflict vs non-conflict MENA countries.** CKM, conservative kidney management.

**Figure 3:**
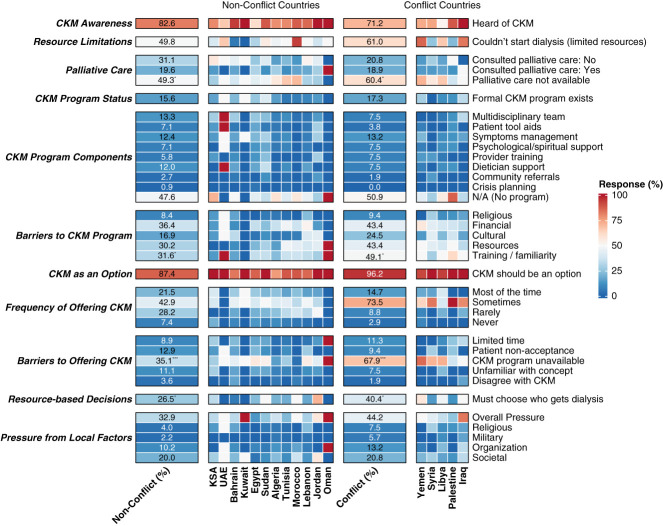
**Heat map of CKM perceptions among nephrologists in the different countries of the MENA region.** The */**/*** indicate significance levels: *=0.05, **=0.01, ***=0.001.

Figure 2 illustrates the awareness, availability, and accessibility of CKM programs in areas of conflict versus nonconflict areas. Awareness of CKM in conflict areas was similar to that in nonconflict areas (OR, 0.55; 95% CI, 0.24 to 1.24; *P* > 0.05) after adjusting for participants' individual and facility characteristics. Participants in conflict zones had less access to palliative care at their institutions (OR, 3.36; 95% CI, 1.30 to 8.68; *P* < 0.05). Only 15.9% of respondents reported having a formal CKM program overall, with a similar proportion in conflict zones compared with nonconflict zones (OR, 0.82; 95% CI, 0.30 to 2.26; *P* > 0.05). Among the 34 formal CKM programs with multidisciplinary teams, only four were located in conflict zones. In conflict zones, participants were more likely to perceive lack of CKM training as the main barrier to its implementation, compared with nonconflict zones (OR, 2.59; 95% CI, 1.24 to 5.38; *P* < 0.05). In addition, nephrologists in conflict zones reported limited access to CKM programs as a significant obstacle when offering CKM to patients, compared with those in nonconflict zones (OR, 3.42; 95% CI, 1.59 to 7.35; *P* < 0.01). Furthermore, more participants in conflict zones tend to face situations where resource limitations forced them to choose who would receive dialysis, compared with those in nonconflict zones (OR, 1.44; 95% CI, 0.67 to 3.07; *P* > 0.05). These data are also provided in tabular form in the Supplemental Material (Supplemental Table 2), while Figure 3 presents the results in granular details across the different MENA countries.

When given the case of a severely morbid and elderly patient who presented with severe KF (Table 2), nephrologists in conflict zones, as compared with those practicing in nonconflict zones, were less likely to consider it morally acceptable not to start dialysis in cases of limited life expectancy (OR, 0.39; 95% CI, 0.17 to 0.88; *P* < 0.05), limited improvements in quality of life (OR, 0.39; 95% CI, 0.18 to 0.85; *P* < 0.05), or when the patient and family refuse dialysis (OR, 0.27; 95% CI, 0.12 to 0.58; *P* < 0.001). Furthermore, nephrologists in conflict zones were also less likely to believe that not initiating dialysis is religiously acceptable when compared with nephrologists in nonconflict areas, even if patient and family agreed to that (OR, 0.41; 95% CI, 0.19 to 0.89; *P* < 0.05).

**Table 2:** Nephrologists' responses when presented by a case of a woman with severe CKD

Questions	Total, *N*^a^ (%)	Nonconflict, *n* (%)	Conflict, *n* (%)
Do you think that not doing dialysis is morally acceptable if the patient and family do not want it? (*N*=252) yes	165 (65)	144 (71)	21 (44)
		—	0.32 (0.17 to 0.62)^c^
		—	0.27 (0.12 to 0.58)^c^
Do you think that not doing dialysis is morally acceptable if the benefit in terms of survival is limited? (*N*=252) yes	172 (68)	147 (72)	25 (52)
		—	0.42 (0.22 to 0.8)^b^
		—	0.39 (0.17 to 0.88)^d^
Do you think that not doing dialysis is morally acceptable if the benefit in terms of quality of life is limited? (*N*=252) yes	155 (62)	133 (65)	22 (46)
		—	0.45 (0.24 to 0.85)^d^
		—	0.39 (0.18 to 0.85)^d^
Do you think that not doing dialysis is religiously acceptable if the patient and family do not want it? (*N*=251) yes	140 (56)	122 (60)	18 (38)
		—	0.42 (0.22 to 0.8)^b^
		—	0.41 (0.19 to 0.89)^d^
Do you think it is important to have a CKM program at your facility? (*N*=252) yes	243 (96)	196 (96)	47 (98)
		—	1.92 (0.23 to 15.72)
		—	1.53 (0.14 to 16.97)
**What percentage of patients with KF who present to you are better treated conservatively without dialysis? (*N*=252)**			
<10%	198 (87)	161 (79)	37 (77)
		—	—
		—	—
10% to <20%	34 (14)	30 (15)	4 (8)
		—	0.58 (0.19 to 1.75)
		—	0.53 (0.15 to 1.92)
>20%	20 (8)	13 (6)	7 (15)
		—	2.34 (0.87 to 6.28)
		—	2.48 (0.62 to 9.88)

A 79-year-old diabetic woman who is blind, with bilateral legs bilateral legs amputations, a stroke 5 years ago that left her with aphasia and swallowing problems problems, on tube feeding. She has progressive CKD. On last visit to her family doctor, she was mildly short of breath, swollen, and had diffuse rales on lung examination. Her urea level was 198 mg/dl (BUN 92 mg/dl), creatinine was 6.9 mg/dl (610 umol/L), and potassium was 5.8 mEq/L. He increased her Lasix dose and referred her to you for dialysis. The patient and the family came to you to understand what is going on and make a decision. CKM, conservative kidney management; KF, kidney failure.

Each row presents the responses in conflict and nonconflict zones. Within each cell, the first line is count (%), the second line is univariable odds ratio (95% confidence interval), and the third line is multivariable odds ratio (95% confidence interval). Odds ratios are in comparison with a baseline of nonconflict regions. For yes/no questions, “no” serves as the baseline, and only an odds ratio for “yes” is listed. For multiple-choice questions, the first option is the baseline, and odds ratios are not provided for this choice. Insuff data mean that the count for that answer choice was too low to calculate an odds ratio.

Variables included in multivariable analysis include: gender, age, employment situation, years of practice, facility location, facility setting, funding entity for dialysis, and access to palliative care.

a Not all 334 participants responded to the above questions. The number of respondents (*N*) for each question is provided alongside the respective question, and *n* represents the count who answered yes within each subcategory: nonconflict and conflict.

b Significance levels=0.01.

c Significance levels=0.001.

d Significance levels=0.05.

## Discussion

In conflict zones, the scarcity of KRT options for patients with advanced kidney disease often makes CKM the default treatment, driven by necessity rather than choice. The higher proportion of part-time nephrologists and reliance on charity-funded dialysis in conflict zones underscores health system fragility and sustainability challenges. Reduced palliative care access directly affects patient quality of life, limiting holistic CKM delivery and compounding ethical dilemmas in end-of-life care. Our study reveals limited awareness of CKM and restricted access to established CKM programs in conflict zones. It also highlights poor access to palliative care, along with lack of training in CKM, as major barriers to CKM implementation in these settings. Besides, our study clearly reflects the moral distress among nephrologists in conflict zones, who face ethical and religious dilemmas when forgoing dialysis, despite its limited benefits.

Conflicts, often occurring in low- and middle-income countries with already fragile health systems, make consistent dialysis care difficult to maintain.^12,13^ All efforts should be exhausted to provide KRT when it extends life and improves quality of life. However, achieving high-quality care for patients on dialysis in areas of conflict is often unattainable.^14^ Studies show that patients on dialysis in these regions experience higher mortality rates, lower access to essential treatments such as erythropoietin-stimulating agents, and higher risks of complications, including infections from catheter dependence.^3,12,15,16^ Amid the harsh realities of war, principles of distributive justice take hold, making dialysis rationing almost inevitable, often leading to reduced session frequency or restricting access to those most likely to benefit.^17^ Under such suboptimal conditions, CKM emerges as a necessary treatment pathway for some patients with advanced kidney disease. Instead of abandoning patients with KF to their fate, health systems have the obligation to provide some level of care, prioritizing symptom management and quality of life.

Armed conflicts can be patchy and protracted, and hence, the availability and accessibility of KRT can vary dramatically, leading to different models of care. Three main scenarios emerge: (*1*) KRT continues with minimal disruption, although this is rare; (*2*) KRT becomes unavailable, making CKM the primary approach with a focus on compassionate care; and (*3*) triage-based care, where resource limitations force prioritizing certain patients for continued KRT while others may have dialysis discontinued or not offered at all. In these cases, CKM serves as the alternative, ensuring patients receive supportive care. These scenarios underscore the urgent need for a structured approach to CKM in conflict zones, upholding the principle of nonabandonment and ensuring humane and dignified care to all patients, regardless of their access to KRT.

Wars often impose unique ethical dilemmas for nephrologists, who must balance the principles of medical ethics such as autonomy, beneficence, and justice while rationing scarce resources such as dialysis.^18,19^ This rationing, often based on utilitarian priorities, represents a profound breach of the ethical principle of nonmaleficence. More nephrologists in conflict zones expressed discomfort with denying dialysis, perceiving it as ethically or religiously unacceptable. This might be driven by heightened religious convictions during dire circumstances, emotional exhaustion from constant exposure to suffering and death, and an overwhelming sense of helplessness. It may also be rooted in deeper cultural and theologic underpinnings, especially in regions where life preservation is a strong moral imperative. This internal conflict—between professional obligation and humanitarian instinct—can generate profound moral distress, especially when the principle of autonomy is overshadowed by the need to ensure equitable resource allocation and treatment choices are driven by resource constraints rather than patient-centered care.^20,21^ This emotional toll is often compounded by the broader impacts of war, which negatively affect their mental health and well-being.^22^ Moral distress might be alleviated if nephrologists were more aware that CKM is an active form of treatment albeit with different ultimate goals under the challenging circumstances.^23^ These goals can be revisited once a crisis is over. Furthermore, promoting procedural justice can mitigate moral distress, by establishing fair, transparent, and consistent policies and guidelines that are tailored to the local context and available resources. This reframes rationing from an isolated moral burden to a shared, system-level ethical response. This underscores the critical role of health care decision makers, including ministries of health, district health administrators, and nongovernmental organizations, in developing such guidance.^24^

Dialysis is often considered the default for patients with ESKD, and there is a reluctance to deviate from the *status quo*. Our study found that fewer nephrologists in conflict zones are aware of CKM, and only four of 66 participants reported multidisciplinary teams for delivering CKM in conflict zones. Similar to studies conducted in other settings, lack of training in CKM emerged as a major obstacle to implementation of CKM in conflict zones.^8,25^ Our study also revealed poor access to palliative care in conflict zones, a major impedance to CKM implementation, that highlights a disconnect between conceptual familiarity and practical infrastructure. Lack of dialysis and palliative care puts patients with advanced CKD at double jeopardy. Patients are often left to suffer physically and psychologically, with ripple effects on their caregivers and communities. Even when palliative care services are available, patients may not be referred in a timely manner if nephrologists are unfamiliar with these services or do not consider palliative care within their scope of responsibility. Barriers to palliative care typically include a shortage of trained specialists and a lack of essential medications including pain medications, antiemetics, diuretics, and antipruritic*.* Access to pain medications, in particular, remains limited, especially in lower-income countries, highlighting the unmet needs to address palliative care gaps in these regions and others facing similar challenges.^26,27^ Addressing these challenges should be a priority in humanitarian efforts, with a focus on training nephrologists, mid-level providers, or nurses to fill gaps in expertise.

Establishing new CKM programs in conflict zones may seem counterintuitive, but many conflicts often persist for years. Moreover, there have been successful initiatives aimed at improving the quality of dialysis in such areas, demonstrating that implementation of new programs is possible even in unstable conditions.^14,28^ While efforts must focus on strategies to optimize dialysis quality and access for those likely to benefit in prolonged conflicts, it is equally important for governments, the World Health Organization, nongovernmental organizations, medical societies, and professional organizations to collaborate at all levels to include CKM in their plans, ensuring all forms of kidney care are continued in the face of emergency. A key challenge is ensuring that health care providers are adequately trained to implement transparent and ethically accountable triage processes in resource-limited settings. This requires not only medical expertise but also a deep understanding of ethical principles, equitable resource allocation, and effective communication with patients and their families. Addressing the unique challenges of conflict zones is essential for developing accessible and sustainable CKM programs. Training is key to this effort, and resources such as the International Society of Nephrology open-access CKM curriculum can support capacity-building.^29^ Table 3 presents the key challenges to implementing CKM and presents proposed strategies to enhance CKM access in conflict zones.

Our study has several limitations. First, the generalizability of our findings may be limited by the respondents' specific backgrounds, which could have influenced their perspectives, particularly in regions where CKM is not widely practiced or where functional CKM programs are lacking. Second, there exists a possibility that health care professionals might have been unable to accurately recall their practice habits or identify specific obstacles related to CKM. Third, we did not consider the income differences between conflict and nonconflict zones, making it unclear whether the observed differences were solely due to conflict status or influenced by the country's income level or baseline health system functioning. Fourth, response rates were higher in conflict zones, which may introduce potential response bias, as participants with more interest or experience in CKM may have been overrepresented, possibly skewing awareness levels and implementation barriers. In addition, small subgroup sizes, particularly in conflict zones, reduced statistical power in some analyses. However, an important strength here is that the results of our study offer novel and unique comparative data on the awareness, acceptance, and implementation of CKM in conflict areas, where research on CKM is nil. Future endeavors should focus on the effectiveness of CKM delivery in conflict zones, understanding cultural and health system-level influences and exploring patient perspectives toward CKM. Furthermore, interpretations regarding moral distress and religious convictions are presented as potential explanations. These are speculative and should be framed as hypotheses for future qualitative research rather than established mechanisms. A deeper understanding of the unique moral and religious discomfort surrounding the withholding of dialysis in war settings is needed. This will require qualitative research approaches, such as in-depth interviews or focus groups, to explore how cultural and religious identities influence ethical decision making under the constraints and pressures of conflict environments.

**Table 3:** Proposed strategies to improve conservative kidney management access in conflict zones

Challenge	Proposed Strategy
Scarcity of KRT options	Promote CKM as a viable and structured alternative, that ensure patients are not abandoned
Limited CKM awareness	Higher CKM education and awareness among nephrologists through educational campaigns, telemedicine, and international support Implement CKM education in local fellowship programs
Poor access to palliative care	Integrate and expand palliative care services within nephrology Dedicate part of the local budget/resources and internal help for palliative care Improve access to essential medications (pain meds, diuretics, *etc.*)
Lack of training in CKM	Build capacity among providers at all levels (doctors, nurses, mid-level) Empower mid-level staff to deliver CKM care Utilize ISN curriculum for CKM Telemedicine help from international expertise
Ethical dilemmas in rationing	Create clear, transparent and consistent guidelines to promote procedural justice Train providers in ethical triage, communication, and decision-making
Emotional and moral distress	Acknowledge CKM as an active, ethically sound and fair treatment alternative when resources are limited
Fragile local health systems	Include CKM in national and humanitarian emergency preparedness plans Integrate CKM into kidney disaster preparedness plans to ensure readiness for future implementation Build CKM programs as resource-light care models
Religious and cultural concerns	Frame CKM in a culturally sensitive way to enhance acceptability
Neglect of CKM in global agendas	Advocate for CKM inclusion in WHO, NGO, and government plans

CKM, conservative kidney management; ISN, International Society of Nephrology; NGO, nongovernmental organization; WHO, World Health Organization.

Our study highlights the unique challenges and moral dilemmas nephrologists face when implementing CKM in conflict zones. Balancing the commitment to patient care with the harsh realities of war necessitates a nuanced approach tailored to the complexities of war. Our study has strong ethical imperative “no patient should be abandoned.” Overcoming barriers such as limited awareness, inadequate training, restricted access to palliative care, and creating ethically sound frameworks are essential to making CKM a viable and ethical pathway for patients with advanced kidney disease in conflict zones. Advocacy for peace and stability is equally crucial to reducing disparities and ensuring equitable access to care.

## Supplementary Material

## Supplemental Material

Supplemental Table 1. Survey in English: attitudes of care providers toward CKM in the MENA region. The translated survey in Arabic and French is available from the authors upon request.

Supplemental Table 2. Perception and attitudes of nephrologists toward CKM programs in the MENA region according to conflict status: univariable and multivariable analysis.
