# Supplementary material for: Conservative Kidney Management in the Middle East and North Africa: A Comparative Study of Conflict and Nonconflict Settings
Source: Clin J Am Soc Nephrol. 2025 Dec 5;21(6):1088–98. doi: 10.2215/CJN.0000000900 (PMC13268373; doi:10.2215/CJN.0000000900)
Supplement: Supplementary file 1 [file cjasn-21-1088-s001.pdf]

ASN American Society of Nephrology | JASN Journal of the American Society of Nephrology | KIDNEY360 Accessing Our World From Every Angle | CJASN Clinical Journal of the American Society of Nephrology

ASN Journal Disclosure Form

As per ASN journal policy, I have disclosed any financial relationships or commitments I have held in the past 36 months as included below. I have listed my Current Employer below to indicate there is a relationship requiring disclosure. If no relationship exists, my Current Employer is not listed.

N. Abuosba reports the following:
Employer: Al-Thawra Hospital; and Advisory or Leadership Role: Director of the Kidney Center, Al-Thawra Hospital; Faculty of Medicine Council and Associate Professor therein.

I understand that the information above will be published within the journal article, if accepted, and that failure to comply and/or to accurately and completely report the potential financial conflicts of interest could lead to the following: 1) Prior to publication, article rejection, or 2) Post-publication, sanctions ranging from, but not limited to, issuing a correction, reporting the inaccurate information to the authors' institution, banning authors from submitting work to ASN journals for varying lengths of time, and/or retraction of the published work.

Name: Najib Wazae Abuosba
Manuscript ID: CJASN-2025-000729R3
Manuscript Title: "Conservative Kidney Management in the Middle East and North Africa: A Comparative Study of Conflict and Non- Conflict Settings.
Date of Completion: November 22, 2025
Disclosure Updated Date: November 22, 2025

ASN American Society of Nephrology
JASN Journal of the American Society of Nephrology
KIDNEY360 Accessing Our World From Every Angle
CJASN Clinical Journal of the American Society of Nephrology

ASN Journal Disclosure Form

As per ASN journal policy, I have disclosed any financial relationships or commitments I have held in the past 36 months as included below. I have listed my Current Employer below to indicate there is a relationship requiring disclosure. If no relationship exists, my Current Employer is not listed.

A. SH. Ali reports the following:
Employer: The Medical City , Baghdad

I understand that the information above will be published within the journal article, if accepted, and that failure to comply and/or to accurately and completely report the potential financial conflicts of interest could lead to the following: 1) Prior to publication, article rejection, or 2) Post-publication, sanctions ranging from, but not limited to, issuing a correction, reporting the inaccurate information to the authors' institution, banning authors from submitting work to ASN journals for varying lengths of time, and/or retraction of the published work.

Name: Ala A. SH. Ali
Manuscript ID: CJASN-2025-000729R1
Manuscript Title: Conservative Kidney Management for Advanced CKD in the MENA Region: A Comparative Study of Conflict and Non-Conflict Settings
Date of Completion: August 13, 2025
Disclosure Updated Date: January 26, 2025

ASN
American Society of Nephrology
JASN
Journal of the American Society of Nephrology
KIDNEY360
Accessing Our World From Every Angle
CJASN
Clinical Journal of the American Society of Nephrology

ASN Journal Disclosure Form

As per ASN journal policy, I have disclosed any financial relationships or commitments I have held in the past 36 months as included below. I have listed my Current Employer below to indicate there is a relationship requiring disclosure. If no relationship exists, my Current Employer is not listed.

K. Allam reports the following:
Employer: University of Minnesota

I understand that the information above will be published within the journal article, if accepted, and that failure to comply and/or to accurately and completely report the potential financial conflicts of interest could lead to the following: 1) Prior to publication, article rejection, or 2) Post-publication, sanctions ranging from, but not limited to, issuing a correction, reporting the inaccurate information to the authors' institution, banning authors from submitting work to ASN journals for varying lengths of time, and/or retraction of the published work.

Name: Krishna C Allam
Manuscript ID: CJASN-2025-000729R2
Manuscript Title: Conservative Kidney Management for Advanced CKD in the MENA Region: A Comparative Study of Conflict and Non-Conflict Settings
Date of Completion: September 15, 2025
Disclosure Updated Date: August 13, 2025

ASN
American Society of Nephrology
JASN
Journal of the American Society of Nephrology
KIDNEY360
Accessing Our World From Every Angle
CJASN
Clinical Journal of the American Society of Nephrology

ASN Journal Disclosure Form

As per ASN journal policy, I have disclosed any financial relationships or commitments I have held in the past 36 months as included below. I have listed my Current Employer below to indicate there is a relationship requiring disclosure. If no relationship exists, my Current Employer is not listed.

A. Almakki reports the following:
Employer: Indiana University Health; and Other Interests or Relationships: Fellow of American College of Physicians (FACP); Fellow of American Society of Nephrology (FASN); Member: Indiana State Medical Association (ISMA), Syrian National Kidney Foundation (SNKF), and Syrian American Medical Society (SAMS); Director of Medical Education at IU Health Arnett; Medical Director of DaVita Home Dialysis Unit of Lafayette.;.

I understand that the information above will be published within the journal article, if accepted, and that failure to comply and/or to accurately and completely report the potential financial conflicts of interest could lead to the following: 1) Prior to publication, article rejection, or 2) Post-publication, sanctions ranging from, but not limited to, issuing a correction, reporting the inaccurate information to the authors' institution, banning authors from submitting work to ASN journals for varying lengths of time, and/or retraction of the published work.

Name: Akram Almakki
Manuscript ID: CJASN-2025-000729R2
Manuscript Title: "Conservative Kidney Management for Advanced CKD in the MENA Region: A Comparative Study of Conflict and Non-Conflict Settings
Date of Completion: September 9, 2025
Disclosure Updated Date: August 4, 2025

ASN
American Society of Nephrology
JASN
Journal of the American Society of Nephrology
KIDNEY360
Accessing Our World From Every Angle
CJASN
Clinical Journal of the American Society of Nephrology

ASN Journal Disclosure Form

As per ASN journal policy, I have disclosed any financial relationships or commitments I have held in the past 36 months as included below. I have listed my Current Employer below to indicate there is a relationship requiring disclosure. If no relationship exists, my Current Employer is not listed.

D. Gunderman reports the following:
Employer: Indiana University School of Medicine

I understand that the information above will be published within the journal article, if accepted, and that failure to comply and/or to accurately and completely report the potential financial conflicts of interest could lead to the following: 1) Prior to publication, article rejection, or 2) Post-publication, sanctions ranging from, but not limited to, issuing a correction, reporting the inaccurate information to the authors' institution, banning authors from submitting work to ASN journals for varying lengths of time, and/or retraction of the published work.

Name: David J. Gunderman
Manuscript ID: CJASN-2025-000729
Manuscript Title: Conservative Kidney Management in Conflict Zones: A Comparative Study in Conflict and Non-Conflict Areas of the MENA Region
Date of Completion: August 12, 2025
Disclosure Updated Date: August 12, 2025

ASN American Society of Nephrology
JASN Journal of the American Society of Nephrology
KIDNEY360 Accessing Our World From Every Angle
CJASN Clinical Journal of the American Society of Nephrology

ASN Journal Disclosure Form

As per ASN journal policy, I have disclosed any financial relationships or commitments I have held in the past 36 months as included below. I have listed my Current Employer below to indicate there is a relationship requiring disclosure. If no relationship exists, my Current Employer is not listed.

T. Hatab has nothing to disclose.

I understand that the information above will be published within the journal article, if accepted, and that failure to comply and/or to accurately and completely report the potential financial conflicts of interest could lead to the following: 1) Prior to publication, article rejection, or 2) Post-publication, sanctions ranging from, but not limited to, issuing a correction, reporting the inaccurate information to the authors' institution, banning authors from submitting work to ASN journals for varying lengths of time, and/or retraction of the published work.

Name: Taha Hatab
Manuscript ID: CJASN-2025-000729R2
Manuscript Title: Conservative Kidney Management for Advanced CKD in the MENA Region: A Comparative Study of Conflict and Non-Conflict Settings
Date of Completion: September 14, 2025
Disclosure Updated Date: September 14, 2025

ASN
American Society of Nephrology
JASN
Journal of the American Society of Nephrology
KIDNEY360
Accessing Our World From Every Angle
CJASN
Clinical Journal of the American Society of Nephrology

ASN Journal Disclosure Form

As per ASN journal policy, I have disclosed any financial relationships or commitments I have held in the past 36 months as included below. I have listed my Current Employer below to indicate there is a relationship requiring disclosure. If no relationship exists, my Current Employer is not listed.

I. Helal reports the following:
Employer: Diaverum AB Holding

I understand that the information above will be published within the journal article, if accepted, and that failure to comply and/or to accurately and completely report the potential financial conflicts of interest could lead to the following: 1) Prior to publication, article rejection, or 2) Post-publication, sanctions ranging from, but not limited to, issuing a correction, reporting the inaccurate information to the authors' institution, banning authors from submitting work to ASN journals for varying lengths of time, and/or retraction of the published work.

Name: Imed Helal
Manuscript ID: CJASN-2025-000729
Manuscript Title: Conservative Kidney Management for Advanced CKD in the MENA Region: A Comparative Study of Conflict and Non-Conflict Settings
Date of Completion: August 13, 2025
Disclosure Updated Date: August 13, 2025

ASN American Society of Nephrology
JASN Journal of the American Society of Nephrology
KIDNEY360 Accessing Our World From Every Angle
CJASN Clinical Journal of the American Society of Nephrology

ASN Journal Disclosure Form

As per ASN journal policy, I have disclosed any financial relationships or commitments I have held in the past 36 months as included below. I have listed my Current Employer below to indicate there is a relationship requiring disclosure. If no relationship exists, my Current Employer is not listed.

M. Hoteit reports the following:
Employer: Bridgeport Hospital/ Yale New Haven Health

I understand that the information above will be published within the journal article, if accepted, and that failure to comply and/or to accurately and completely report the potential financial conflicts of interest could lead to the following: 1) Prior to publication, article rejection, or 2) Post-publication, sanctions ranging from, but not limited to, issuing a correction, reporting the inaccurate information to the authors' institution, banning authors from submitting work to ASN journals for varying lengths of time, and/or retraction of the published work.

Name: Mayssaa Hoteit
Manuscript ID: CJASN-2025-000729R1
Manuscript Title: Conservative Kidney Management for Advanced CKD in the MENA Region: A Comparative Study of Conflict and Non-Conflict Settings
Date of Completion: August 13, 2025
Disclosure Updated Date: August 13, 2025

ASN American Society of Nephrology
JASN Journal of the American Society of Nephrology
KIDNEY360 Accessing Our World From Every Angle
CJASN Clinical Journal of the American Society of Nephrology

ASN Journal Disclosure Form

As per ASN journal policy, I have disclosed any financial relationships or commitments I have held in the past 36 months as included below. I have listed my Current Employer below to indicate there is a relationship requiring disclosure. If no relationship exists, my Current Employer is not listed.

S. Koubar reports the following:
Employer: University of Minnesota; and Research Funding: Otsuka; Amgen; Alexion; NKF.

I understand that the information above will be published within the journal article, if accepted, and that failure to comply and/or to accurately and completely report the potential financial conflicts of interest could lead to the following: 1) Prior to publication, article rejection, or 2) Post-publication, sanctions ranging from, but not limited to, issuing a correction, reporting the inaccurate information to the authors' institution, banning authors from submitting work to ASN journals for varying lengths of time, and/or retraction of the published work.

Name: Sahar Koubar
Manuscript ID: 2025-000729
Manuscript Title: Conservative Kidney Management for Advanced CKD in the MENA Region: A Comparative Study of Conflict and Non-Conflict Settings
Date of Completion: August 12, 2025
Disclosure Updated Date: August 12, 2025

ASN
American Society of Nephrology
JASN
Journal of the American Society of Nephrology
KIDNEY360
Accessing Our World From Every Angle
CJASN
Clinical Journal of the American Society of Nephrology

ASN Journal Disclosure Form

As per ASN journal policy, I have disclosed any financial relationships or commitments I have held in the past 36 months as included below. I have listed my Current Employer below to indicate there is a relationship requiring disclosure. If no relationship exists, my Current Employer is not listed.

V. Luyckx reports the following:
Employer: University of Zurich; Consultancy: World Health Organization, Ethics and NCDs; Honoraria: Honoraria for one Grand Rounds and for moderation of one NIH meeting.; Honoraria from one talk for a biotech company donated directly from the company to the Red Cross Childrens Hospital fund in Cape Town; Patents or Royalties: Royalties as Editor of Brenner and Rector's The Kidney, Elsevier; and Advisory or Leadership Role: Unpaid - past Chair Advocacy Working Group, ISN; Unpaid - Chair Ethics Committee ERA; Unpaid - president European Kidney Health Alliance.

I understand that the information above will be published within the journal article, if accepted, and that failure to comply and/or to accurately and completely report the potential financial conflicts of interest could lead to the following: 1) Prior to publication, article rejection, or 2) Post-publication, sanctions ranging from, but not limited to, issuing a correction, reporting the inaccurate information to the authors' institution, banning authors from submitting work to ASN journals for varying lengths of time, and/or retraction of the published work.

Name: Valerie A. Luyckx
Manuscript ID: CJASN-2025-000729
Manuscript Title: Conservative Kidney Management for Advanced CKD in the MENA Region: A Comparative Study of Conflict and Non-Conflict Settings
Date of Completion: August 13, 2025
Disclosure Updated Date: August 13, 2025

ASN American Society of Nephrology | JASN Journal of the American Society of Nephrology | KIDNEY360 Accessing Our World From Every Angle | CJASN Clinical Journal of the American Society of Nephrology

ASN Journal Disclosure Form

As per ASN journal policy, I have disclosed any financial relationships or commitments I have held in the past 36 months as included below. I have listed my Current Employer below to indicate there is a relationship requiring disclosure. If no relationship exists, my Current Employer is not listed.

M. Sayegh has nothing to disclose.

I understand that the information above will be published within the journal article, if accepted, and that failure to comply and/or to accurately and completely report the potential financial conflicts of interest could lead to the following: 1) Prior to publication, article rejection, or 2) Post-publication, sanctions ranging from, but not limited to, issuing a correction, reporting the inaccurate information to the authors' institution, banning authors from submitting work to ASN journals for varying lengths of time, and/or retraction of the published work.

Name: Mohamed H. Sayegh
Manuscript ID: CJASN-2025-000729R2
Manuscript Title: Conservative Kidney Management for Advanced CKD in the MENA Region: A Comparative Study of Conflict and Non-Conflict Settings
Date of Completion: September 11, 2025
Disclosure Updated Date: August 5, 2025

ASN American Society of Nephrology
JASN Journal of the American Society of Nephrology
KIDNEY360 Accessing Our World From Every Angle
CJASN Clinical Journal of the American Society of Nephrology

ASN Journal Disclosure Form

As per ASN journal policy, I have disclosed any financial relationships or commitments I have held in the past 36 months as included below. I have listed my Current Employer below to indicate there is a relationship requiring disclosure. If no relationship exists, my Current Employer is not listed.

A. Shebani has nothing to disclose.

I understand that the information above will be published within the journal article, if accepted, and that failure to comply and/or to accurately and completely report the potential financial conflicts of interest could lead to the following: 1) Prior to publication, article rejection, or 2) Post-publication, sanctions ranging from, but not limited to, issuing a correction, reporting the inaccurate information to the authors' institution, banning authors from submitting work to ASN journals for varying lengths of time, and/or retraction of the published work.

Name: Abdulhafid Ali Shebani
Manuscript ID: CJASN-2025-000729R1
Manuscript Title: Conservative Kidney Management for Advanced CKD in the MENA Region: A Comparative Study of Conflict and Non-Conflict Settings,"
Date of Completion: August 13, 2025
Disclosure Updated Date: August 13, 2025

ASN Journal Disclosure Form

As per ASN journal policy, I have disclosed any financial relationships or commitments I have held in the past 36 months as included below. I have listed my Current Employer below to indicate there is a relationship requiring disclosure. If no relationship exists, my Current Employer is not listed.

R. Yamout has nothing to disclose.

I understand that the information above will be published within the journal article, if accepted, and that failure to comply and/or to accurately and completely report the potential financial conflicts of interest could lead to the following: 1) Prior to publication, article rejection, or 2) Post-publication, sanctions ranging from, but not limited to, issuing a correction, reporting the inaccurate information to the authors' institution, banning authors from submitting work to ASN journals for varying lengths of time, and/or retraction of the published work.

Name: Rana Yamout
Manuscript ID: CJASN-2025-000729
Manuscript Title: Conservative Kidney Management in Conflict Zones: A Comparative Study in Conflict and Non-Conflict Areas of the MENA Region
Date of Completion: August 13, 2025
Disclosure Updated Date: August 13, 2025
