# Supplementary material for: Conservative Kidney Management in the Middle East and North Africa: A Comparative Study of Conflict and Nonconflict Settings
Source: Clin J Am Soc Nephrol. 2025 Dec 5;21(6):1088–98. doi: 10.2215/CJN.0000000900 (PMC13268373; doi:10.2215/CJN.0000000900)
Supplement: Supplementary file 2 [file cjasn-21-1088-s002.pdf]

Supplementary Material
Table S1. Survey in English: Attitudes of Care Providers towards Conservative Kidney Management in the Middle East and North Africa Region

1-Demographics:

What is your gender?	❑ Male<br>❑ Female
How old are you?	____ years
Which option describes your current employment situation?	❑ Have a full-time employment<br>❑ Have a part-time employment<br>❑ Retired<br>❑ I prefer not to answer
Number of years in nephrology practice after graduation	❑ < 5<br>❑ 5-10<br>❑ 10-15<br>❑ 15-20<br>❑ >20
Are you a pediatric nephrologist?	❑ Yes<br>❑ No

2-Facility Characteristics:

Where is your facility located?	❑ Rural/village<br>❑ Suburban<br>❑ Urban (City)
Which country do you practice in?	❑ Algeria<br>❑ Egypt<br>❑ Jordan<br>❑ Iraq<br>❑ Lebanon<br>❑ Libya<br>❑ Morocco<br>❑ Palestine (West bank and Gaza)<br>❑ Syria<br>❑ Sudan<br>❑ Tunisia<br>❑ KSA<br>❑ Bahrain

	❑ Oman<br>❑ Kuwait<br>❑ United Arab Emirates<br>❑ Yemen<br>❑ Qatar
What is your facility setting?	❑ Academic<br>❑ Private<br>❑ Governmental<br>❑ Military<br>❑ Non-profit<br>❑ Others (Please specify)
What is the main funding entity for dialysis at your facility? (please check the most common funding source)	❑ Governmental<br>❑ Insurance<br>❑ Private (self-Pay)<br>❑ Charity<br>❑ Other, please specify
What is the main funding entity for pre-dialysis care at your facility? (please check the most common funding source)	❑ Governmental<br>❑ Insurance<br>❑ Private (self-Pay)<br>❑ Charity<br>❑ Other, please specify
What is the main funding entity for palliative care at your facility? (please check the most common funding source)	❑ Governmental<br>❑ Insurance<br>❑ Private (self-Pay)<br>❑ Charity<br>❑ Other, please specify
Are the patients responsible for paying for their dialysis sessions?	❑ Not at all<br>❑ Partially<br>❑ Fully
Does your institution have a palliative care practice?	❑ Yes<br>❑ No
Do you have a nephrology social worker in your dialysis unit?	❑ Yes<br>❑ No

3-Please, answer these questions to the best of your knowledge:

1- What percentage of your patients need the assistance of other people to bring them to dialysis?	❑ <5%<br>❑ 5-<10%<br>❑ 10-<15%<br>❑ 15-<20%<br>❑ 20-<25%<br>❑ >25%<br>❑ Other(specify):
2- What percentage of your patients need a sitter/companion during dialysis sessions?	❑ <5%<br>❑ 5-<10%<br>❑ 10-<15%<br>❑ 15-<20%<br>❑ 20%-<25%<br>❑ >25%<br>❑ Other(specify):
3- What percentage of your patients have a poor functional status i.e dependent on others in their daily activities?	❑ <5%<br>❑ 5-<10%<br>❑ 10-<15%<br>❑ 15-<20%<br>❑ 20%-<25%<br>❑ >25%<br>❑ Other(specify):
4- What percentage of your dialysis patients are expected to die within 3 months? (numerical scroll down)	❑ 1-3%<br>❑ 4-6%<br>❑ 7-9%<br>❑ >10%
5- Have you heard of conservative kidney management as an option to treat advanced CKD without Kidney Replacement Therapy? Medically advised or chosen **Conservative kidney Management is defined as "planned, holistic, patient-centered care for patients with CKD stage 5, that**	❑ Yes<br>❑ No

**includes interventions to delay progression of kidney disease and minimize complications but focuses predominantly on symptom management and psychological, social, cultural and spiritual support <u>but does not include dialysis,"</u>**	
6- Have you faced a situation where you couldn't start dialysis on a patient because of limited resources in the past 5 years?	❑ Yes<br>❑ No

4- Please answer these questions regarding your practice?

1-Do you conduct a formal Quality of life questionnaire for your dialysis patients:	❑ Yes<br>❑ No
2-Have you ever consulted palliative care services on your **<u>outpatient</u>** dialysis patients **(Not during hospitalization)?**	❑ Yes<br>❑ No<br>❑ Palliative care is not available at my institution
3-Do you have a formal CKM program at your institution?	❑ Yes<br>❑ No
4a-If you have a formal CKM program, what components are available in you program: (check all that applies)	❑ Multidisciplinary team<br>❑ Patient tool aids<br>❑ Symptoms management<br>❑ Psychological, cultural, spiritual support<br>❑ Health care provider training in CKM<br>❑ Dietician support<br>❑ Community referrals<br>❑ Crisis planning<br>❑ I don't have CKM program

4b-If you don't have a formal CKM program, please indicate whether you believe each of these aspects is a barrier to having such a program:	❑ Religious y/n<br>❑ Financial y/n<br>❑ Cultural y/n<br>❑ Resources y/n<br>❑ Training / familiarity in CKM y/n<br>❑ Others, specify
5-Do you think CKM should be an option when discussing kidney replacement therapy?	❑ Yes<br>❑ No
6-If you answer yes to above, how often do you offer CKM to your patients?	❑ Most of the time<br>❑ Sometimes<br>❑ Rarely<br>❑ Never
7-If you answer never to the above question, what is usually the barrier?	❑ Limited time in clinic<br>❑ Patient will insist on dialysis and seek care with another nephrology provider<br>❑ CKM program is not available in my clinic<br>❑ Not familiar with the concept<br>❑ I don't agree with CKM
8-Do *you* have to choose who gets dialysis treatment and who does not because of limited resources?	❑ Yes<br>❑ No
9-Have you ever experienced pressure from local factors (such as religious, military, organization, societal, etc.) when making decisions related to patient care?	❑ Yes<br>❑ No<br><br>If yes, please specify:<br>-Religious<br>-Military<br>-Organization<br>-Societal<br>-Others

5-Case Study: The following is a hypothetical case of a patient; Please answer the questions related to it

79-year-old diabetic woman who is blind, history of bilateral legs amputations, a stroke five years ago that left her with aphasia and swallowing problems. On tube feeding. She has progressive CKD. On last visit to her family doctor, she was mildly short of breath, was swollen, had diffuse rales on lung examination. Her Urea level was 198 mg/dl (BUN 92 mg/dl), creatinine 6.9 mg / dl (610 umol/L), the potassium was 5.8 mEq /L. He increased her Lasix dose and referred her to you for dialysis. The patient and the family came to you to understand what is going on and make a decision.

What would you do?	❑ Recommend that the patient gets a catheter placed and start dialysis ASAP<br><br>❑ Explain to the patient and family that the benefit from dialysis in her condition is very limited and suggest that they consider foregoing it<br><br>❑ Tell the patient and her family it is wrong to dialyze her and you will not do that
If this patient did not go on dialysis for any reason, what would you do?	❑ Suggest to the family to take her home<br>❑ Send her back to the PCP to continue managing her<br>❑ Refer her to an established program for conservative kidney management in your area

6-Please answer these questions about your attitude towards dialysis initiation:

Do you think that not doing dialysis is morally acceptable if the patient and family do not want it?	❑ Yes<br>❑ No
Do you think that not doing dialysis is morally acceptable if the benefit in terms of survival is limited?	❑ Yes<br>❑ No
Do you think that not doing dialysis is morally acceptable if the benefit in terms of Quality of Life (QOL) is limited?	❑ Yes<br>❑ No
Do you think that not doing dialysis is religiously acceptable if the patient and family do not want it?	❑ Yes<br>❑ No
Do you think it is important to have a conservative kidney management program at your facility?	❑ Yes<br>❑ No
What percentage of the CKD V patients who present to you are better treated conservatively without dialysis?	❑ 5-<10%<br>❑ 10-<15%<br>❑ 15-<20%<br>❑ 20-<25%<br>❑ >25%
Feel free to add a comment you would like us to know about your attitude towards conservative kidney management (CKM)	

CKD chronic kidney disease; PCP primary care physician, QoL Quality of life; CKM conservative kidney management;

Table S2: Perception and attitudes of nephrologists towards CKM programs in the MENA region according to Conflict Status: Univariable and Multivariable analysis^^

Questions	Total $N^{\#}$ (%)	Non-conflict n (%)	Conflict n (%)
Have you heard of conservative kidney management? (yes, N=312)	251 (80)	209 (83)<br>-<br>-	42 (71)<br>0.52 (0.27, 1.00)*<br>0.55 (0.24, 1.24)
Have you faced a situation where you couldn't start dialysis on a patient because of limited resources in the past 5 years? (yes, N=312)	162 (52)	126 (50)<br>-<br>-	36 (61)<br>1.58 (0.88, 2.81)<br>1.80 (0.91, 3.54)
Have you ever consulted palliative care services on your outpatient dialysis patients? (N=278)			
No	81 (29)	70 (31.10)<br>-<br>-	11 (21)<br>-<br>-
Yes	54 (19)	44 (20)<br>-<br>-	10 (19)<br>1.45 (0.57, 3.69)<br>1.41 (0.43, 4.57)
Palliative Care not available in my institution	143 (51)	111 (49)<br>-<br>-	32 (60)<br>1.83 (0.87, 3.87)<br>3.36 (1.3, 8.68)*
Do you have a formal CKM program at your institution? (yes, N=276)	44 (16)	35 (16)<br>-<br>-	9 (17)<br>1.13 (0.51, 2.53)<br>0.82 (0.30, 2.26)
If you have a formal CKM program, what components are available in your program? (N=276)			

Multidisciplinary team	34 (12)	30 (13) - -	4 (8) 0.53 (0.18, 1.58) 0.41 (0.11, 1.54)
Patient tool aids	18 (7)	16 (7) - -	2 (4) Insuff data Insuff data
Symptoms management	35 (13)	28 (12) - -	7 (13) 1.07 (0.44, 2.60) 0.85 (0.29, 2.51)
Psychological, cultural, spiritual support	20 (7)	16 (7) - -	4 (8) 1.07 (0.34, 3.33) 1.13 (0.28, 4.53)
Health care provider training in CKM	17 (6)	13 (6) - -	4 (8) 1.33 (0.42, 4.26) 0.8 (0.18, 3.66)
Dietician support	31 (11)	27 (12) - -	4 (8) 0.6 (0.2, 1.79) 0.47 (0.13, 1.66)
Community referrals	7 (3)	6 (3) - -	1 (2) Insuff data Insuff data
Crisis planning	2 (0.07)	2 (1) Insuff data Insuff data	0 (0.0) Insuff data Insuff data
N/A (No CKM program)	134 (49)	107 (48) - -	27 (51) 1.15 (0.63, 2.08) 1.26 (0.61, 2.59)
If you don't have a formal CKM program, please indicate whether you believe each of these aspects is a barrier to having such a program (N=278)			

Religious	24 (9)	19 (8)<br>-<br>-	5 (9)<br>1.13 (0.4, 3.18)<br>0.67 (0.18, 2.53)
Financial	105 (38)	82 (36)<br>-<br>-	23 (43)<br>1.34 (0.73, 2.45)<br>1.19 (0.57, 2.5)
Cultural	51 (18)	38 (17)<br>-<br>-	13 (25)<br>1.6 (0.78, 3.27)<br>1.32 (0.54, 3.2)
Resources	91 (33)	68 (30)<br>-<br>-	23 (43)<br>1.77 (0.96, 3.27)<br>1.96 (0.92, 4.17)
Training / familiarity in CKM	97 (35)	71 (32)<br>-<br>-	26 (49.1)<br>2.09 (1.14, 3.83)*<br>2.59 (1.24, 5.38)*
Do you think CKM should be an option when discussing kidney replacement therapy? (yes, N=274)	244 (89)	194 (87)<br>-<br>-	50 (96)<br>3.61 (0.83, 15.64)<br>3.14 (0.59, 16.63)
How often do you offer CKM to your patients? (yes, N=197)			
Most of the times	40 (20)	35 (22)<br>-<br>-	5 (15)<br>-<br>-
Sometimes	95 (48)	70 (43)<br>-<br>-	25 (74)<br>2.5 (0.88, 7.09)<br>2.4 (0.61, 9.45)
Rarely	49 (25)	46 (28)<br>-<br>-	3 (9)<br>0.46 (0.1, 2.04)<br>0.83 (0.13, 5.16)

Never	13 (7)	12 (7)<br>-<br>-	1 (3)<br>Insuff data<br>Insuff data
What is usually the barrier for not offering CKM to your patients? (yes, N=278)			
Limited time in clinic	26 (9)	20 (9)<br>-<br>-	6 (11)<br>1.31 (0.50, 3.44)<br>1.32 (0.41, 4.3)
Anticipated non-acceptance from the patient	34 (12)	29 (13)<br>-<br>-	5 (9)<br>0.7 (0.26, 1.91)<br>0.55 (0.16, 1.86)
CKM program is not available at my practice	115 (8)	79 (35)<br>-<br>-	36 (68)<br>3.91 (2.07,7.41)***<br>3.42 (1.59, 7.35)**
Not familiar with the concept	29 (10)	25 (11)<br>-<br>-	4 (8)<br>0.65 (0.22, 1.96)<br>0.67 (0.20, 2.28)
I don’t agree with CKM	9 (3)	8 (4)<br>-<br>-	1 (2)<br>Insuff data<br>Insuff data
Do you have to choose who gets dialysis treatment and who does not because of limited resources? (yes, N=271) Yes response?	79 (29)	58 (27)<br>-<br>-	21 (40)<br>1.88 (1, 3.53)*<br>1.44 (0.67, 3.07)
Have you ever experienced pressure from local factors when making decisions related to patient care? (yes, N=278) Yes response?			
Local Press	95 (35)	72 (33)<br>-<br>-	23 (44)<br>1.62 (0.87, 3)<br>1.96 (0.93, 4.14)
Religious	13 (5)	9 (4)<br>-<br>-	4 (8)<br>1.96 (0.58, 6.62)

			1.83 (0.39, 8.63)
Military	8 (3)	5 (2)<br>-<br>-	3 (6)<br>2.64 (0.61, 11.41)<br>5.13 (0.79, 33.32)
Organization	30 (11)	23 (10)<br>-<br>-	7 (13.2)<br>1.34 (0.54, 3.3)<br>2.26 (0.75, 6.8)
Societal	56 (20)	45 (20)<br>-<br>-	11 (21)<br>1.05 (0.5, 2.2)<br>1.15 (0.46, 2.84)

^^Each row presents the responses in conflict and non-conflict zones. Within each cell, the first line is count (%), the second line is univariable odds ratio (95% confidence interval), and the third line is multivariable odds ratio (95% CI). Odds ratios are in comparison to a baseline of non-conflict regions. For yes/no questions, "no" serves as the baseline, and only an odds-ratio for "yes" is listed. For multiple-choice questions, the first option is the baseline, and odds ratios are not provided for this choice. Insuff data means that the count for that answer choice was too low to calculate an odds ratio. Abbreviations: CKD, Chronic Kidney Disease; CKM, Conservative Kidney Management. Significance levels: *=0.05, **=0.01, ***=0.001. Variables included in multivariable analysis include: gender, age, employment situation, years of practice, facility location, facility setting, funding entity for dialysis, access to palliative care.

[#] Not all 334 participants responded to the above questions. The number of respondents (N) for each question is provided alongside the respective question and n represents the count who answered yes within each subcategory: non-conflict and conflict.
